# Decision-making by cancer patients and the role of a counselling facility for complementary and alternative medicine: a cohort study

**DOI:** 10.1007/s00432-022-04178-9

**Published:** 2022-07-11

**Authors:** Vanessa Hack, Lena Josfeld, Jutta Hübner, Christian Keinki, Jens Büntzel

**Affiliations:** 1 Klinik für Innere Medizin II, Universitätsklinikum Jena, Jena, Germany; 2 Klinik für HNO-Erkrankungen, Südharzklinikum, Nordhausen, Germany; 3 Christeserstraße, 98547 Kühndorf, Germany

**Keywords:** Shared decision-making (SDM), Patient participation in health care, Cancer, Informed decision, Evidence-based patient choice, Medical advice/consultation, Complementary and alternative medicine (CAM)

## Abstract

**Objective:**

The aim of this cohort study was to gain insight on influencing factors on the decision-making process in conventional medicine compared to complementary and alternative medicine (CAM).

**Methods:**

A standardized questionnaire was distributed among cancer patients who attended the counselling facility for CAM of a German university hospital in 2020.

**Results:**

Fifty patients (30.3%) returned the questionnaire. After counselling on CAM, most patients made a decision in CAM but also in conventional medicine. Thus, the focus on informed decision-making during counselling on CAM had a strong effect on the decision-making process in conventional medicine. Patients reporting good support also reported making decisions together with physicians and relatives. Moreover, after counselling on CAM, patients reported being more satisfied with their decision in both settings afterwards.

**Conclusion:**

Information on CAM which focuses on informed decision-making, supports patient’s ability to understand and weigh risks and benefits of treatments, supports shared decision-making and enables patients to transfer these competences also to decisions on conventional medicine. So counselling on CAM may further decision-making competences in cancer patients. This encourages patients to engage in shared decision-making and increases patient’s satisfaction with decisions.

## Introduction

In the year 2021, almost 500,000 patients in Germany were informed about their cancer diagnosis (Robert Koch-Institut (Hrsg) 2017). Many patients experience their disease as “a burden associated with emotional distress” (Teng et al. 2014), as for many patients, this diagnosis can be linked with the fear of death (Sharpe et al. 2018). Suddenly, the disease determines life and questions private and professional plans. Therefore, the disease and its consequences can already diminish their quality of life (Grassi et al. 2009; Dehkordi et al. 2009).

With a diagnosis as complex as cancer (Sverdlov 2016), there is often more than one treatment option. Therefore, the diagnosis of cancer is associated with numerous important decisions. Every patient has the legally guaranteed right to have a say in their own cancer therapy treatment. It is assumed that people, who are better informed about their illness and the different treatment options, can take on more self-responsibility for their health (Sharpe et al. 2018). Besides, participation as an equal partner in the decision-making is an opportunity for patients to play an active part during therapy and to feel more self-determined (Kroker et al. 2019).

While in conventional medicine, the different treatment options are based on scientific evidence and guidelines, in complementary and alternative medicine (CAM), patients decide by themselves or discuss options with naturopaths, non-medical practitioners or other lay-people. Moreover, shared decision-making is the preferred model in conventional treatment (Linke et al. 2015; Lang et al. 2017), while to our knowledge, no data exist on decision-making in CAM.

CAM is no topic on which patients have to decide in the context of other treatment decisions. CAM is voluntary. In contrast, most patients do not receive evidence-based information in their centres. Moreover, most physicians do not support decision-making on CAM. As a consequence, patients have to look for information and ways to decide for themselves.

Evidence-based counselling on CAM (Huebner et al. 2013) therefore focuses on providing evidence-based information in the methods, but also helping patients to define their aims and to make decisions, to integrate their relatives and to pursue their aims. Thus, CAM counselling also aims at patient empowerment.

Accordingly, the aim of this pilot study was to gain a first insight into influencing factors on the decision-making process in conventional medicine and in CAM. We examined whether patients were confronted with a decision before visiting the counselling facility for CAM and whether participants made a decision afterwards. Another important aim of the survey was to find out what role the counselling facility for CAM played in the decision-making process, especially whether patients found the advice helpful. One of the main aspects was the sufficient support from others in the decision-making process as an influencing factor. In addition, we analysed the type of decision-making regarding the people involved in the decision-making process, the awareness of knowledge about treatment options, their advantages and disadvantages as well as their side effects and the satisfaction with the decision afterwards dependent on the patients’ support. We examined the various influencing factors in a comparison between conventional medicine and CAM.

The results of this study may help strengthen the patient's ability to make decisions in medical context and thus improve patient-centred cancer care.

## Methods

### Study design and sample

This survey was a cohort study conducted from November 2020 to April 2021 at a German university cancer centre. We asked all patients who attended the counselling facility on Complementary Medicine in 2020 via post to participate in an anonymised, standardized questionnaire with 21 questions. Questionnaires were sent by post to 165 patients. Participants were informed that the participation in the study was voluntary and anonymous.

### Questionnaire

The survey consisted of a standardized questionnaire which was developed by the working group Prevention and Integrative Oncology of the German Cancer Society based on a questionnaire on complementary and alternative medicine (CAM) developed by this group (Huebner et al. 2014). Patients were asked to answer these questions once in case they already had made a decision on conventional cancer therapy, once when they had made a decision on CAM. In the latter case, we asked some additional questions which parts of the counselling had been helpful for them. Furthermore, we asked for their satisfaction with their decision.

The final questionnaire (see online Supplemental Material) contained four categories and 21 questions:

1. Demographic data (gender, age, federal state, type of cancer, treatment situation).
2. Decision-making in conventional medicine (decisional conflict scale (Légaré et al. 2010; O’Conner 1993) (DCS) and decisional regret scale (O’Conner 1996) (DRS) plus additional items).
3. Decision-making in CAM [DCS (Légaré et al. 2010; O’Conner 1993) and DRS (O’Conner 1996) plus additional items].
4. Rating of elements of the counselling facility for CAM for decision-making.

In the section “decision-making in conventional medicine” and “decision-making in CAM”, we asked participants about their decision-making after medical advice, type of decision-making, state of knowledge, circumstances of the decision, satisfaction with the decision and medical advice.

We used closed questions (“Yes”, “No”, “I don’t know”) to assess whether a patient was facing a decision before the consultation and whether they did make a decision afterwards. Concerning the people who were involved in the decision-making, participants were asked to choose between “alone by me”, “by my doctor”, “by my relatives”, “by me and my doctor”, “by me and my relatives”, “by me, my relatives and my doctor”, “by someone else”. We used the DCS (Légaré et al. 2010; O’Conner 1993) and DRS (O’Conner 1996) which both employ a five-point Likert scale from “strongly disagree”, “disagree”, “neither agree nor disagree”, “agree” to “strongly agree” to collect information about the state of knowledge (treatment options and their advantages, disadvantages and side effects), the support during the decision-making process, and satisfaction with decision/regrets about decision. Participants were then asked if the medical advice at the counselling facility was helpful for decision-making. Patients who marked, that they had not made a decision, were asked for their reasons.

### Participants

We addressed all cancer patients visiting the counselling facility for complementary and alternative medicine (CAM) of the outpatient ward of the Department for Haematology and Medical Oncology of the University Hospital Jena in Germany in 2020.

### Setting

In the counselling facility, patients who are looking for advice on CAM get a structured counselling on integrative oncology, starting with a summary of the cancer disease, former and actual treatment, experiences of the patient in this trajectory including side effects and communication with the physicians. Next, the questions of the patient on CAM are collected, and each topic is discussed considering aims and evidence on benefits and harms.

### Informed consent

Informed consent was given by all participants by filling in the questionnaire.

### Ethical approval

This study was performed in line with the principles of the Declaration of Helsinki. The study was approved by the ethic committee of the university hospital Jena (Approval No. 2020-1976_Bef).

### Statistics

Data collection and the statistical analyses were conducted in IBM SPSS Statistics for Windows (IBM SPPS Statistics, Armonk, NY, USA, version 28.0). The scores of the DCS (Légaré et al. 2010; O’Conner 1993) and DRS (O’Conner 1996) were calculated according to the user manuals. During analyses, missing data were handled by pairwise deletion.

Several regressions were run to look for factors influencing decision-making. Association between two nominal or one nominal and one ordinal variable was tested via Fisher’s exact test which is more robust than the chi-square test when cell frequencies in the cross tables fall below five. We also calculated Cramer’s-V as a measure of the association between two nominal variables. Furthermore, we used Kruskal–Wallis tests to determine whether or not there is a statistically significant difference between the medians of three or more independent groups. Associations between age as a metric variable and various other aspects were tested for via univariate analyses of variance (ANOVA) or Kruskal–Wallis tests where requirements for an ANOVA were not met; Eta-squared was determined for effect size.

*P* values < 0.05 were considered as significant.

## Results

### Demographic data

Questionnaires were sent to 165 patients by mail. Of 165 patients, who were asked to participate in the survey, 50 answered our questionnaire (return rate 30.3%). Sixty per cent of the participants disclosing gender were women, and 40% were men. The average age of all participants was 60.7 years.

As expected, the most common tumours were breast cancer, haematologic cancer (lymphoma, multiple myeloma), prostate cancer and gastrointestinal tumours.

Twenty-eight percent of the patients that visited the counselling facility for CAM were post-cancer treatment, and 68% were before or under cancer treatment.

For further information about demographic data see Table 1.

**Table 1:** Demographic data (*N* = 50)

	Total	%
Gender		
Male	20	40%
Female	30	60%
Age		
< 30 years	1	2%
31–50 years	7	14%
51–70 years	31	62%
71–80 years	10	20%
> 80 years	1	2%
Type of cancer		
Breast cancer	12	24%
Haematological cancer	8	16%
Prostate cancer	6	12%
Gastrointestinal carcinoma	14	28%
Ovarian cancer	2	4%
Other^a^	7	14%
No answer	1	2%
Treatment situation		
Patient before or under cancer treatment	34	68%
Patient post-cancer treatment	14	28%
No answer	2	4%

^a^Renal cell carcinoma (RCC), Bladder Cancer, Lung Cancer, Malignant melanoma, more than one type of tumour or tumour not specified

### Decision facing and making and the helpfulness of the counselling for CAM (Fig. 1)

**Fig. 1:**
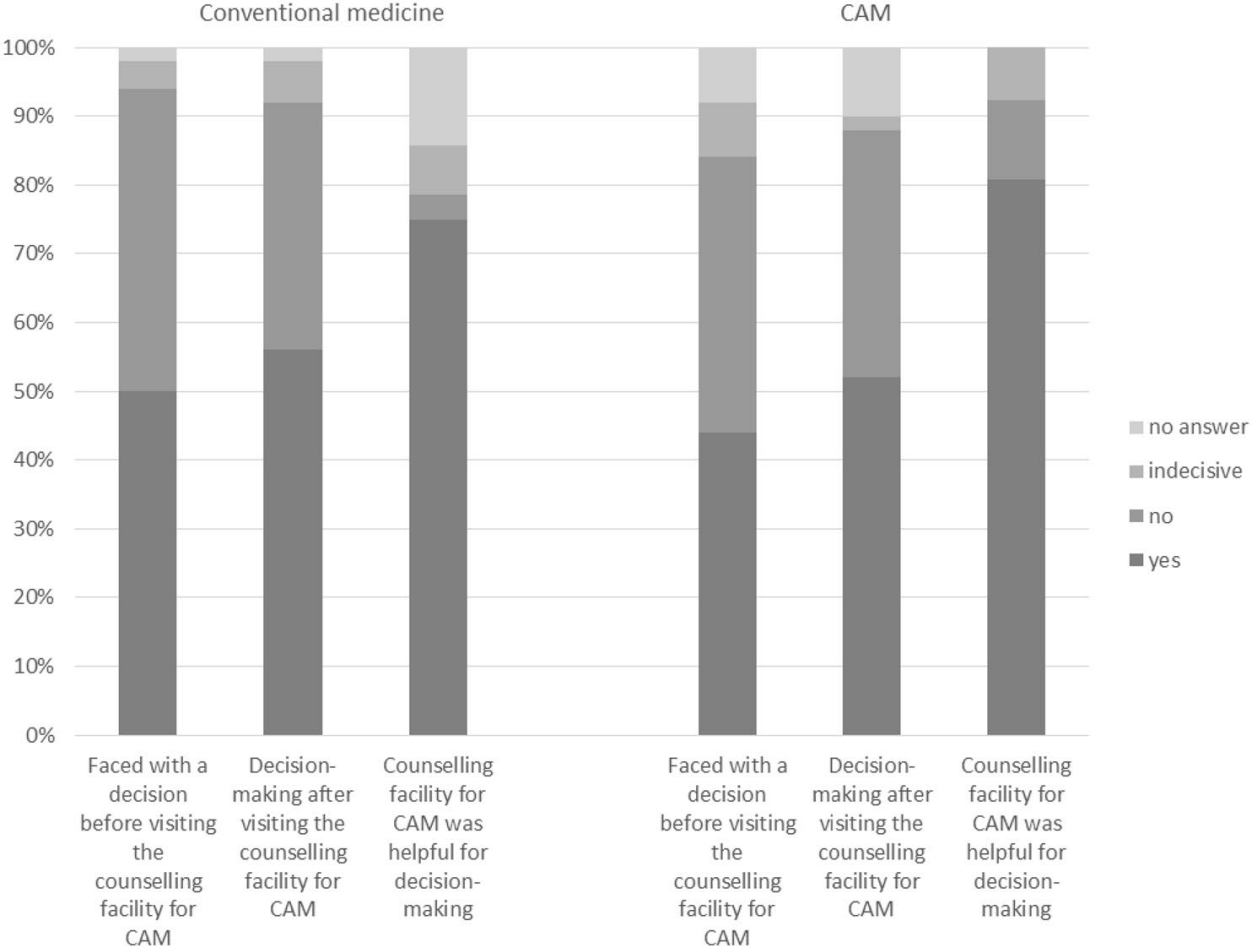
Facing a decision, making a decision, and helpfulness of the counselling for CAM (*N* = 100, last question *N* = 54)

Fifty per cent of the patients were aware of a decision they faced in conventional medicine before visiting the counselling facility for CAM. Forty-four per cent were aware of facing a decision in CAM. After visiting the counselling facility for CAM, 56.0% made a decision regarding their conventional cancer therapy and 52.0% made a decision on CAM. Seventy-five percent of the patients who made a decision in conventional medicine and 80.8% of the patients who made a decision in CAM found the medical advice given by the counselling facility for CAM helpful for their decision-making. In conventional medicine 4.1% and in CAM 6.7% did not make a decision after consulting, although they felt they were facing a decision before. 12.3% of people in conventional medicine and 8.9% in CAM made a decision afterwards, although they stated that they were not faced with a decision before visiting the counselling centre.

For further information see Fig. 1.

### People involved in decision-making (Fig. 2)

**Fig. 2:**
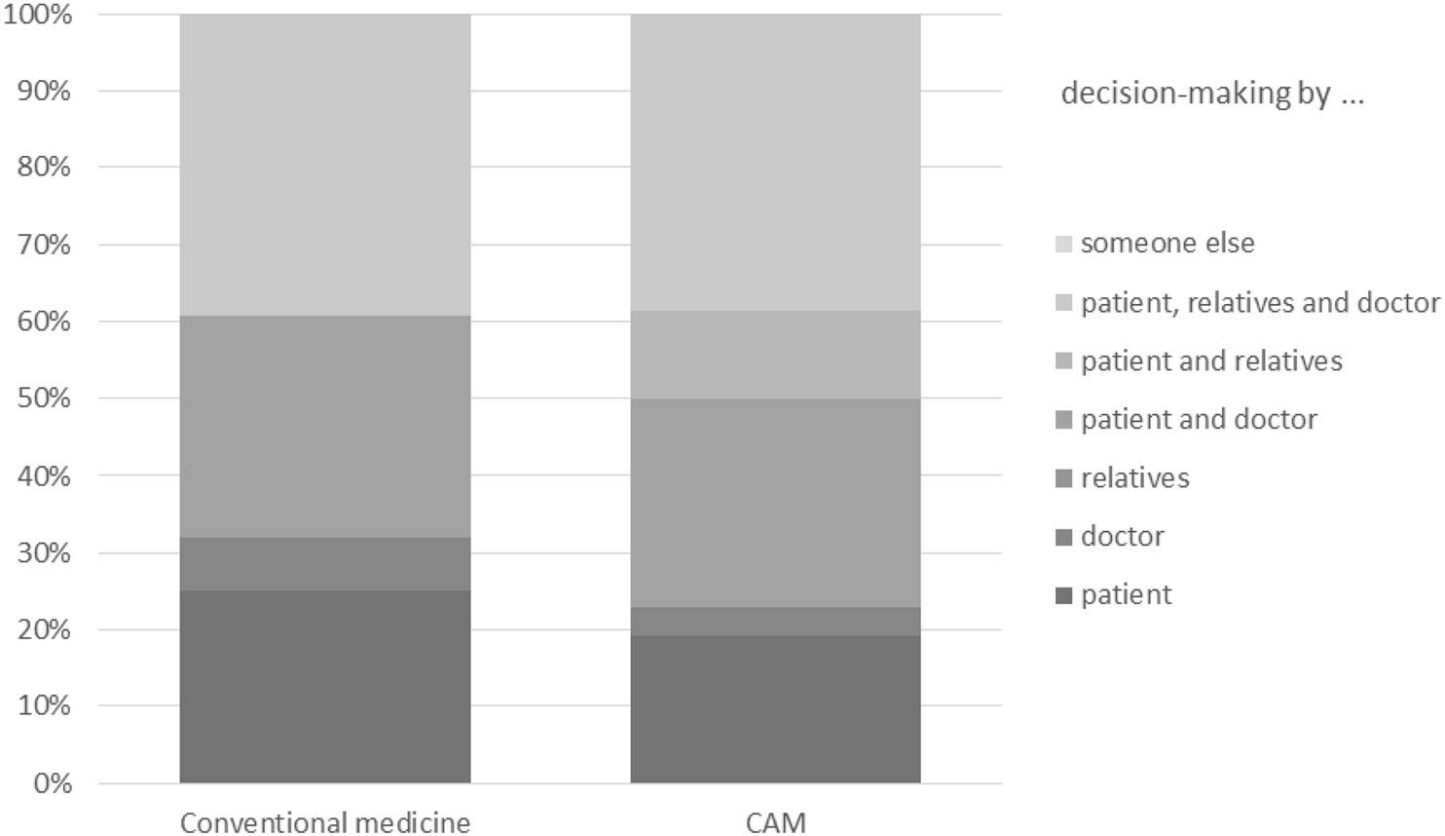
People involved in decision-making (*N* = 54)

Shared decision-making (SDM) was reported in conventional medicine by 28.6% and in CAM by 26.9%. In contrast to SDM, the decision was made solely by the doctor in 7.1% of the cases in conventional cancer therapy and by 3.8% in CAM. Twenty-five per cent of the patients in conventional medicine and 19.2% of the patients in CAM made the decision by themselves. In conventional medicine 39.3% and in CAM 38.5% of the patients made their decision together with their relatives and their physicians. In conventional medicine nobody and in CAM 11.5% of the participants decided together with their relatives. No decision was made solely by relatives or other people in either group.

For further information see Fig. 2.

### Decisional conflict and regret (Table 2)

**Table 2:** Decisional conflict and regret

	Average	SD	Minimum	Maximum
Decisional conflict scale (DCS) (Légaré et al. 2010; O’Conner 1993)				
Total score				
Conventional medicine	22.6	12.3	1.6	45.3
CAM	22.8	13.9	1.6	45.3
Uncertainty (regarding decisions)				
Conventional medicine	31.1	24.9	0.0	83.3
CAM	43.2	16.6	25.0	75.0
Informed (about available options)				
Conventional medicine	25.3	18.9	0.0	58.3
CAM	48.6	17.9	25.0	100.0
Values clarity				
Conventional medicine	16.7	15.9	0.0	0.0
CAM	42.6	17.2	25.0	100.0
Effective decision				
Conventional medicine	20.2	12.7	0.0	50.0
CAM	16.2	14.5	0.0	37.5
Decisional regret scale (DRS) (O’Conner 1996)				
Total score				
Conventional medicine	14.7	15.4	0.0	55.0
CAM	9.1	11.5	0.0	45.0

The total score of decisional conflict of our study population according to the DCS has an average of 22.6 in conventional medicine (SD: 12.3, Min: 1.6, Max: 45.3) and 22.8 in CAM (SD: 13.9, Min: 1.6, Max: 45.3), with lower values indicating less decisional conflict. For subscores see Table 2.

The decisional regret of our study population according to the DRS has an average of 14.7 in conventional medicine (SD: 15.4, Min: 0.0, Max: 55.0) and 9.1 in CAM (SD: 11.5, Min: 0.0, Max: 45.0), with lower values indicating less regret.

We found no correlation between the scales’ overall and the subscores on the one hand and variables age, type of cancer, treatment situation and type of decision-making on the other hand. The only exception was a gender effect on the subscore “values clarity” in the field of CAM: Women were less clear about their values and which benefits and values matter most to them (MD = 50) than men (MD = 37.5) (exact Mann–Whitney *U* test: *U* = 30.5, *p* = 0.047). According to Cohen (Cohen 1992), the effect is moderate at *r* = 0.43.

In order to find indications of a possible relevance of individual aspects of the DCS and DRS, we then performed analyses on the single items.

### Information and advice for decision-making, support during decision-making and self-rating of the decision by the patients (Fig. 3)

**Fig. 3:**
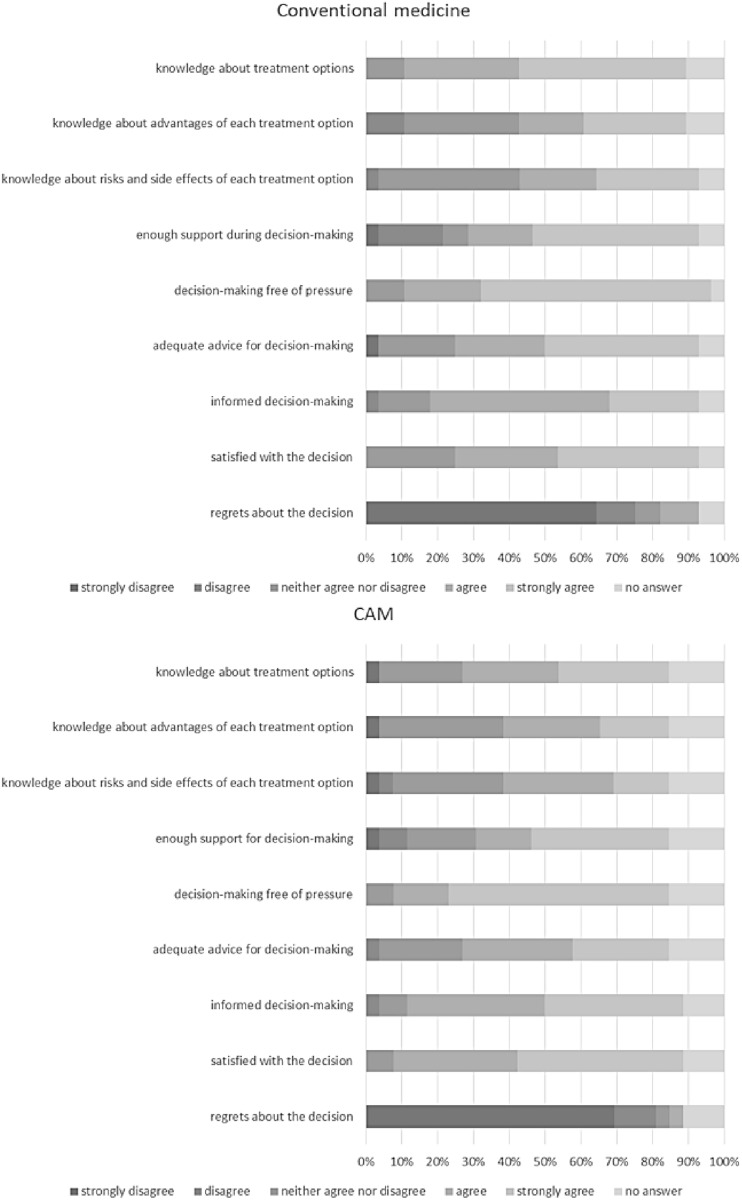
Information and advice for decision-making, support during decision-making and self-rating of the decision by the patients (*N* = 54)

In conventional medicine, concerning the item “I know what treatment options I have”, 46.4% of the participants strongly agreed and 32.1% agreed. With regard to the aspect “I know the advantages of each treatment option”, 28.6% of the patients strongly agreed and 17.9% agreed. Regarding knowledge on risks and side effects of treatment options, 28.6% of the participants strongly agreed and 21.4% agreed. To the item “I got enough support during decision-making”, 46.4% of the patients strongly agreed and 17.9% agreed. Concerning the aspect decision-making free of pressure, 64.3% strongly agreed and 21.4% agreed. Adequate advice for decision-making was strongly agreed to by 42.9% of the participants and agreed to by 25.0%. Furthermore, patients rated the extent to which they would agree that they made an informed decision after visiting the counselling facility for CAM, to which 25.0% strongly agreed and 50.0% agreed. Regarding the statement “I am satisfied with my decision”, 39.3% of the patients strongly agreed and 28.6% agreed. Nobody strongly agreed with the statement “I regret my decision”. 64.3% of the patients strongly disagreed.

Regarding CAM, 30.8% of the participants strongly agreed and 26.9% agreed that they are aware of the different treatment options. With regard to their knowledge about the advantages of each treatment option, 19.2% of the patients strongly agreed and 26.9% agreed. The awareness of knowledge about risks and side effects of treatment options was strongly agreed to by 15.4% and agreed to by 30.8%. To the item “I got enough support during decision-making”, 38.5% of the patients strongly agreed and 15.4% agreed. Concerning the aspect decision-making free of pressure, 61.5% strongly agreed and 15.4% agreed. Adequate advice for decision-making was strongly agreed to by 26.9% of the participants and agreed to by 30.8%. The statement “I made an informed decision” was strongly agreed to and agreed to each by 38.5% of the participants. Regarding the statement “I am satisfied with my decision”, 46.2% strongly agreed and 34.6% agreed. Nobody strongly agreed with the statement “I regret my decision”. 69.2% strongly disagreed.

For further information see Fig. 3.

### Correlations (Table 3)

**Table 3:** Correlations

Conventional medicine	CAM
**Facing a decision and making a decision after visiting the counselling facility for CAM (Fisher’s exact test,** ***p*** **= 0.000,** ***n*** **= 49; Cramer’s** ***V*** **= 0.737)** Age and type of decision-making (*F* (3) = 2.601, *p* = 0.075, *n* = 27 **Type of decision-making and enough support during decision-making (Fisher’s exact test,** ***p*** **= 0.035,** ***n*** **= 26; Cramer’s** ***V*** **= 0.495)** **Age of participants and their satisfaction with the decision afterwards (Kruskal–Wallis test** **:** ***H*** **(2) = 9.363,** ***p*** **= 0.009)** **Treatment situation and satisfaction with the decision (Fisher’s exact test,** ***p*** **= 0.043,** ***n*** **= 25; Cramer’s** ***V*** **= 0.493)** Amount of knowledge about risks and side effects of each treatment option and amount of satisfaction with decision (Fisher’s exact test, *p* = 0.076, *n* = 25) Decision-making free of pressure or manipulation of other people’s desires and satisfaction with decision (Fisher’s exact test, *p* = 0.075, *n* = 26) **Helpfulness of the counselling facility for CAM for decision-making and satisfaction with decision (Fisher’s exact test = 6.919,** ***p*** **= 0.013,** ***n*** **= 22; Cramer’s** ***V*** **= 0.459)** Amount of adequate advice for decision-making and amount of satisfaction with decision (Kruskal–Wallis test: *H*(2) = 5.581, *p* = 0.061)	Type of tumour and facing a decision (Fisher’s exact test, *p* = 0.055, *n* = 45) **Confronted with decision before and making a decision after visiting the counselling facility for (Fisher’s exact test,** ***p*** **= 0.000,** ***n*** **= 45; Cramer’s** ***V*** **= 0.583)** Helpfulness of the counselling facility for CAM for decision-making and amount of support while decision-making (Fisher’s exact test, *p* = 0.071, *n* = 22) **Amount of support while making the decision and amount of knowledge about the risks and side effects of different treatment options (Fisher’s exact test,** ***p*** **= 0.014,** ***n*** **= 22; Cramer’s** ***V*** **= 0.654)** Amount of knowledge about risks and side effects of different treatment options and feeling of an informed decision-making (Fisher’s exact test, *p* = 0.056, *n* = 22) Amount of knowledge about risks and side effects of treatment options for decision-making and amount of satisfaction with decision (Fisher’s exact test, *p* = 0.056, *n* = 22) **Enough support during decision-making and satisfaction with decision (Fisher’s exact test,** ***p*** **= 0.001,** ***n*** **= 22; Cramer’s** ***V*** **= 0.683)** **Informed decision-making and satisfaction with decision (Fisher’s exact test,** ***p*** **= 0.001,** ***n*** **= 22; Cramer’s** ***V*** **= 0.642)** **Adequate advice for decision-making and satisfaction with the decision (Kruskal–Wallis test** **:** ***H*** **(2) = 6.909,** ***p*** **= 0.032)**

The correlations marked in bold are the significant correlations (*p* < 0.05)

In conventional medicine as well as in CAM, we found no associations between the state of knowledge and age, gender, type of cancer or treatment situation.

In conventional medicine and CAM, we found no difference in being aware of a pending decision or decision-making with respect to gender, age and treatment situation, but we found a tendency towards patients with gastrointestinal tumours more frequently reporting facing a decision on CAM than patients with other types of tumour (Fisher’s exact test, *p* = 0.055, *n* = 45).

In both, conventional medicine and CAM, people who were aware of being confronted with a decision before the consultation, made a decision afterwards significantly more often than people who were not aware of an impending decision before the consultation (conventional medicine: Fisher’s exact test, *p* = 0.000, *n* = 49; CAM: Fisher’s exact test, *p* = 0.000, *n* = 45). Cramer’s *V* indicates a strong (*V* = 0.737) correlation in the field of conventional medicine and a medium (*V* = 0.583) correlation in the field of CAM.

For CAM, patients who found the advice helpful tended to experience better support while making the decision. (Fisher’s exact test, *p* = 0.071, *n* = 22).

In the field of conventional medicine, there was a tendency towards an association between the age of participants and the type of decision-making, in which younger patients made their decisions more frequently together with their relatives and their physicians, whereas older participants rather made their decisions together with their doctors and without their relatives (*F*(3) = 2.601, *p* = 0.075, *n* = 27). There was a significant association between the type of decision-making in conventional medicine and the aspect “enough support during decision-making” (Fisher’s exact test, *p* = 0.035, *n* = 26). Patients stating they had good support were most likely to make decisions together with their doctor and relatives.

In the field of CAM, there was a medium-sized positive correlation (Cramer’s *V* = 0.654) between the amount of support while making the decision and the amount of knowledge about the risks and side effects of different treatment options (Fisher’s exact test, *p* = 0.014, *n* = 22). Furthermore, we found a tendency of a positive correlation between the amount of knowledge about risks and side effects of different treatment options and the feeling of making an informed decision (Fisher’s exact test, *p* = 0.056, *n* = 22).

In conventional medicine, we found no associations between patients’ satisfaction with their decision and gender, type of cancer or treatment situation, but we found a significant association between the age of the participants and their satisfaction with the decision. Younger patients were significantly more often completely satisfied with their decision, while older patients were more likely to be partially satisfied (Kruskal–Wallis test: *H*(2) = 9.363, *p* = 0.009). Moreover, we determined a moderate association between treatment situation and satisfaction with the decision (Fisher’s exact test, *p* = 0.043, Cramer’s *V* = 0.493, *n* = 25). Patients after conventional cancer therapy were more satisfied with their decision than patients before or under cancer treatment. None of the respondents stated that they were completely or partly unsatisfied with their conventional medical decision, regardless of their treatment situation. There is a trend of a positive association between the amount of knowledge about risks and side effects of each treatment option and the amount of satisfaction with the decision afterwards (Fisher’s exact test, *p* = 0.076, *n* = 25), as well as between decision-making free of pressure or manipulation of other people’s desires and the satisfaction with the decision afterwards (Fisher’s exact test, *p* = 0.075, *n* = 26). Further analyses showed a positive correlation in the field of conventional medicine between the variables “advice in the counselling facility for CAM was helpful for decision-making” and satisfaction with the decision (Fisher’s exact test = 6.919, *p* = 0.013, *n* = 22) as well as a tendency of a positive association between the amount of adequate advice for decision-making and the amount of satisfaction with the decision (Kruskal–Wallis test: *H*(2) = 5.581, *p* = 0.061).

In CAM, we found no associations between patients’ satisfaction with their decision and age, gender, type of cancer or treatment situation, but there was also a tendency of a positive correlation between the amount of knowledge about risks and side effects of treatment options for decision-making and the amount of satisfaction with the decision (Fisher’s exact test, *p* = 0.056, *n* = 22). Furthermore, we found a highly significant and moderate correlation between the two aspects “enough support during decision-making” and “satisfaction with the decision” (Fisher’s exact test, *p* = 0.001, *n* = 22; Cramer’s *V* = 0.683). Patients who felt enough support were more satisfied. We also detected a moderate association between the variables “informed decision-making” and “satisfaction with the decision” (Fisher’s exact test, *p* = 0.001, *n* = 22; Cramer’s *V* = 0.642) as well as between “adequate advice for decision-making” and “satisfaction with the decision” (Kruskal–Wallis test: *H*(2) = 6.909, *p* = 0.032). Patients were more satisfied if they felt they made an informed decision and if they felt they received adequate advice.

For further information about correlations see Table 3.

## Discussion and conclusion

### Discussion

To our knowledge, this cohort study is the first to assess the influencing factors on the decision-making process by cancer patients in conventional medicine compared to CAM.

One finding of our study is that patients facing any decision before the consultation were likely to make this decision after visiting the counselling facility for CAM.

In relation to this, it is interesting that in both areas, conventional medicine and CAM, some patients were unaware that they were faced with a decision before the consultation. This could be due to the fact that not all patients perceive a decision as such if they choose not to change anything. Evidence has also shown that doctors and patients have very different perceptions of whether a decision was made in a conversation or not (Leppin et al. 2015). Patients may not have been able to perceive the decision because when talking to the doctor it sounded as if there was no alternative option and they did not have the opportunity to say “No”, as they did not get any information on other treatment options and/or palliative care.

On the other hand, in both areas, a few participants did not make a decision after the consultation, although they were confronted with a decision before. Owing to the fact that the decision-making process can take some time (Fischhoff and Broomell 2020; Guo 2008), it could be that the patients were still preparing the decision at the time of the survey, so they were still in the information collection and evaluation phase and wanted to obtain further opinions, e.g. a second opinion from another oncologist. It is also conceivable that these participants were still deliberating which decision to make finally, considering the risks and benefits of the treatment options. Physicians should engage in minimizing concerns and help patients to make decisions that they are least likely to regret by giving health education and support (Ozdemir and Finkelstein 2018). Participants could also have lacked confidence in the counselling (Fischhoff and Broomell 2020) or felt overwhelmed after the counselling. While there is a wide variety of potential reasons, only very few participants answered the question why they have not yet made the decision. Moreover, some patients prefer the physician to make the decision all alone (Efficace et al. 2014; Gaston and Mitchell 2005). These aspects should be examined more closely in further studies.

In conventional medicine, patients with good support by others made shared decisions more often with the doctor and their relatives. Participants who had less support were more likely to make their treatment decisions alone or together with the doctor. In particular, younger participants made decisions more often together with the doctor and their relatives, whereas older patients made decisions primarily with the doctor without their relatives. It has been described before, that older patients are more likely to make instant decisions than younger patients (Meyer et al. 2007), which indirectly shows that decision-making in older adults is more independent of others. Interestingly, these aspects were only given in conventional medicine. This could be because the support in conventional medicine is more relevant for patients than in CAM.

With regard to satisfaction with the decisions, younger patients were more likely to be completely or rather satisfied with their decisions, while older patients were more likely to be partly satisfied. This could possibly be related to the fact that younger patients felt more support from relatives during the decision-making process. Support is a very important factor influencing the decision-making process and the subsequent satisfaction (Milky and Thomas 2020; Wei et al. 2020; Nakayama et al. 2020).

Another factor influencing satisfaction was the treatment situation. Survivors after conventional treatment, during follow-up, were more satisfied with their decisions regarding their cancer therapy. One reason might be that with the decision being longer ago, the patient realizes the positive effects, while the side effects decrease. This strongly reduces the doubts on the treatment decision.

In CAM, patients, who found the advice on CAM helpful, stated more often that they received sufficient support during decision-making and enough advice to make the decision. This is relevant as the support seems to be a very important factor influencing decision-making and satisfaction in both areas. Patients confronted with a decision benefit from the medical advice in the information collection phase of the decision-making process which makes them more satisfied with their decision afterwards.

Correspondingly, participants with sufficient support reported more often that they had a good knowledge about risks and side effects of their choices in CAM. Accordingly, patients with good knowledge about risks and side effects were more likely to feel they made an informed decision. In both, conventional medicine and CAM, knowledge about risks and side effects influenced satisfaction with decisions. Balanced information on the benefits as well as the risks and side effects in this patient-centred counselling facility is presented in an understandable and comprehensible way for patients, so that they are able to rank the various treatment options and then select the best alternative. There is no evidence that provision of additional information leads to adverse reactions by patients, on the contrary, poor transmission of information and low understandability seems to lower the satisfaction and compliance (Ley 1982).

In case of a decision being made while carefully considering the pros and cons of each option, patients will be able to deal better with the side effects of a treatment option as they seem most justifiable to them.

In conventional medicine and CAM, patients reporting an informed decision were more likely to be satisfied with their decisions.

In fact, the counselling facility for CAM had a positive effect on the satisfaction with the decisions in CAM and conventional medicine, especially by preparing patients to make decisions in a field they themselves are responsible for. Besides information, patients learn more about decision-making and improve their competences. This may provide them with a template to improve their ability to play an active role in the decision-making process, which in turn increases competences in decision-making and the satisfaction with decision afterwards.

Moreover, in conventional medicine, patients who made the decision without pressure were more satisfied with their decisions. Above all, pressure can be psychologically stressful. This includes time pressure or pressure to act, but also the pressure exerted on the decision-maker by other people. When making decisions, it is relevant for the affected patients to take their time (Beers et al. 2017) and make decisions carefully. Pressure could make patients feel overwhelmed, the ability to consider decisions and the associated consequences could decrease. Thus, satisfaction with the decision could decrease, raising doubts as to whether the decision was made optimally. As CAM is no subject which needs pressure, patients may learn to take time for information, deliberation and decision-making. Not only in CAM but also in conventional medicine, oncologists should therefore elicit and respect patients’ preferences and goals (Beers et al. 2017) and give patients time for reflection, whenever possible, in order to improve satisfaction and adherence (Morris and Schulz 1992).

The most important limitation of the study is the rather low number of participants. The low response rate is in part related to the cancer-caused death of patients; others may have been reluctant to reflect on a difficult decision-making process, while for some, the timespan between the consultation and the data collection phase may have been too long.

Participants may not be representative because of a selection bias, as we addressed only cancer patients that visited the counselling facility for CAM at the university hospital in Jena. This is the only hospital in Jena which also provides medical care to the rural surroundings. Participants came from different federal states from Germany, but most of them from Thuringia.

Furthermore, we only addressed patients in ambulatory care, so our results may not be valid for inpatients.

Another important limitation is the subjective perception of what a decision is and the subjective rating of the counselling facility’s role for decision-making. As a result, the patients may have said that they did not make a decision—even if they did—because they did not perceive it as one.

It is also possible that especially patients who were satisfied with the given advice responded more often to our survey which in turn limits the representativeness. Therefore, the role of the counselling facility for CAM may have been overlooked, overestimated or underestimated by the patients in this study.

### Conclusion

Advice in the counselling facility plays an important role in the patients’ decision-making process. It supports patients confronted with a decision in their individual decision-making by decision-making skills training. This may improve patient-centred cancer care.

Along with the feeling of making an informed decision, sufficient advice increases the patient's satisfaction with the decision. Our study clearly shows that counselling positively influences satisfaction. Likewise, support from a doctor and/or relatives also has a positive effect on a patient’s satisfaction.

Patients are more satisfied if they have good knowledge about risks and side effects of each treatment option before making a final decision. It is therefore important that doctors provide detailed and understandable information with a focus on the risks and side effects of each treatment option. Relatives also seem to contribute to improving the patient's level of knowledge. A recommendation from the doctor to actively involve relatives in the decision-making process is advisable. Care must be taken to ensure that no pressure is exerted on the patient, as this has a negative effect on the patient's satisfaction with the decision.

### Practice implications

Information on complementary medicine which focuses on informed decision-making, supports patients’ ability to understand and weigh risks and benefits of treatments, supports shared decision-making and enables patients to transfer these competences also to decisions on conventional medicine. Doctors should keep this in mind in order to provide the patient with a corresponding amount of information and to give them special support in the decision-making process (Elwyn et al. 2012). Specific training for patients on how to make personally fitting and satisfying decisions could also be beneficial (Efficace et al. 2014). Training these competences as well as the support from a physician and/or relatives in general has a positive effect on a patients’ satisfaction with decision-making. The involvement of a counselling facility and patients’ relatives in the decision-making process and in the concept of shared decision-making is encouraged in order to strengthen the ability in making more quality decisions, to improve the support during decision-making and to increase patients’ satisfaction with decisions, whereby age, gender, treatment situation, information needs and other “individual differences in decision-making competence” (Talukdar et al. 2018) of the patients should be considered.

## Data Availability

The datasets generated during and/or analysed during the current study are available from the corresponding author on reasonable request.
